# Non-bulky Lesions in Human DNA: the Ways of Formation, Repair, and Replication

**Published:** 2017

**Authors:** A.V. Ignatov, K.A. Bondarenko, A.V. Makarova

**Affiliations:** Institute of Molecular Genetics of Russian Academy of Sciences, Kurchatov sq. 2, Moscow, 123182 , Russia; Department of Molecular Biology, Faculty of Biology, Moscow State University, Leninskie Gory 1, bldg. 12, Moscow, 119991, Russia

**Keywords:** DNA damage, DNA repair, DNA translesion synthesis

## Abstract

DNA damage is a major cause of replication interruption, mutations, and cell death. DNA damage is removed by several types of repair processes. The involvement of specialized DNA polymerases in replication provides an important mechanism that helps tolerate persistent DNA damage. Specialized DNA polymerases incorporate nucleotides opposite lesions with high efficiency but demonstrate low accuracy of DNA synthesis. In this review, we summarize the types and mechanisms of formation and repair of non-bulky DNA lesions, and we provide an overview of the role of specialized DNA polymerases in translesion DNA synthesis.

## INTRODUCTION

Numerous lesions occur daily in the DNA of living organisms, either spontaneously or caused by various chemical and physical factors, such as free radicals, ultraviolet (UV) and ionizing radiation, cell metabolites, and chemical carcinogens. In addition, DNA lesions occur during physiological cellular reactions (e.g*., *intermediates of DNA repair and hypermutation of immunoglobulin genes).

Chemical carcinogens, such as acrolein, cisplatin, benzo[α]pyrene, aromatic amines, nitrosamines, and UV radiation result in the preferential formation of bulky adducts, intra- and interstrand crosslinks, which substantially distort the geometry of the framework of DNA [1]. These lesions are eliminated from genomic DNA mainly by nucleotide excision repair (NER) [2, 3]. The unrepaired bulky lesions significantly inhibit the activity of the high-fidelity DNA polymerases (Pols) that are specifically involved in genomic DNA replication and are guided by the strict geometric complementarity during nucleotide incorporation [4-6]. The accumulation of these lesions in dividing cells results in replication interruption, chromosomal aberrations, and cell death.

Spontaneous DNA lesions and those formed during cell metabolism or resulting from free radical attacks are mostly non-bulky. The main groups of non-bulky DNA lesions include apurinic/apyrimidinic sites (AP sites), oxidized and some alkylated nucleotide derivatives, as well as lesions caused by deamination of DNA bases. Base excision repair (BER) is the key mechanism for the elimination of such lesions. The BER machinery has been discussed in detail in several recent reviews [7-9]. Although non-bulky lesions have a lesser effect on the DNA structure, they also alter the function of DNA replication enzymes by causing DNA copying errors and blocking replication.

In the present review, we systematize the main pathways of formation, repair, and replication of non-bulky DNA lesions and discuss the role of the specialized human Pols that ensure efficient, although often error-prone, translesion DNA synthesis.

## TYPES OF DNA LESIONS AND THEIR FORMATION

**Apurinic and apyrimidinic sites**

Apurinic and apyrimidinic sites (AP sites), the most frequent DNA lesions, play a crucial role in mutagenesis induced by genomic DNA damage. The average number of AP sites that emerge daily in a mammalian cell is 9,000–14,000 [10, 11]. Most AP sites are a result of the spontaneous hydrolysis of the N-glycosidic bond in deoxyribonucleotides that occurs under physiological conditions at a considerably high rate [12]. The cleavage rate of purine bases (apurination) is more than 10-fold higher than that of pyrimidine bases [11]. The glycosidic bond cleavage is also catalyzed by DNA glycosylases during BER [13]. Finally, the formation of AP sites is a key stage in the hypermutation of immunoglobulin genes in mammals [14, 15].

AP sites simultaneously exhibit high mutagenic and cytotoxic properties. Since no canonical hydrogen bonds can be formed with AP sites, many Pols are paused or incorporate dAMP opposite the lesion (*Table*) [16-19]. Incorporation of dAMP opposite an AP site is energetically more favourable [20]. In the lagging DNA strand, AP sites inhibit strand displacement synthesis by Pol δ during replication, thereby disrupting Okazaki fragment maturation [21]. Unrepaired AP sites cause transcription termination and are responsible for the high frequency of mutagenesis in *Saccharomyces cerevisiae* [22]. AP sites in human DNA are predominantly recognized and cleaved by AP endonuclease 1 (APE1), yielding single-strand breaks [23, 24]. It should be noted that many other proteins have recently been found to play a role in the alternative pathways of APE1-independent repair of AP sites: subunits of the Ku protein that is a component of DNA-dependent protein kinase (DNA-PK) [25-27], tyrosyl-DNA phosphodiesterase I (TDP1) [28, 29], and poly(ADP-ribose) polymerase 1 (PARP1) [30]. The alternative pathways of AP site repair can act as auxiliary DNA repair mechanisms. The functions of Ku and TDP1 proteins in the repair of an AP site have been discussed more thoroughly in previous reviews [31, 32].

**Table:** Efficiency and fidelity of replication opposite non-bulky DNA lesions by human Pols

Damage		Pol	Replication efficiency	Preferential incorporation of nucleotides
AP site		Replicative Pols		
		Pol α	+ [18, 189]	dAMP [18] dAMP ≈ dTMP [189]
		Pol δ	+ [17]; +++ [189] (Pol δ/PCNA); inhibits strand displacement activity [21]	dAMP [189]
		Pol ε	- [16]	
		Specialized Pols		
		Pol η	+++ [189, 191] ++++ [194, 196]	dAMP [191, 192, 196] dAMP ≈ dTMP [189] dAMP ≈ dGMP [194]
		Pol ι	++ [189, 197, 199] ++++ [196] and in cooperation with ScPol ζ [197]	dTMP [189, 196] dGMP [197, 198, 199]
		Pol κ	+ [189, 196]	dCMP ≥ dAMP [189] dAMP [196]
		PrimPol	- [233, 237, 242]; + [238]; ++++ [234]	dAMP ≈ deletions [238] deletions [234]
Deamination	U	Replicative Pols		
		Pol δ	++++ [60] (ScPol δ)	dAMP [60] (ScPol δ)
		Pol ε	++++ [60] (ScPol δ)	dAMP [60] (ScPol δ)
		Specialized Pols		
		Pol ι	++++ [200] (U, 5-oh-U and 5,6-DHU)	dGMP opposite U, 5-oh-U and 5,6-DHU [200]
		PrimPol	++++ [237]	dAMP [237]
	hypoxanthine	Replicative Pols		
		Pol α	+++ [62]	dCMP [62]
		Specialized Pols		
		Pol η	++++ [62]	dCMP [62]
		Pol κ	++++ [62]	dCMP [62]
Oxidation	8-oxo-G	Replicative Pols		
		Pol α	+ [40]	dAMP [40]
		Pol δ	+ [17, 40]	dAMP [40]
		Specialized Pols		
		Pol η	++++ [195, 201] and [193] in vivo +++ [42]	dCMP [195] dAMP ≈ dCMP [42] dAMP [201]
		Pol ι	+ [198]; ++ [201]; ++++ [200]	dCMP [199–201]
		Pol κ	+++ [201]	dAMP [201]
		PrimPol	++++	dCMP [236, 240]
	TG	Replicative Pols		
		Pol α	- [44, 192]	dAMP [192]
		Specialized Pols		
		Pol η	+++ [192]	dAMP [192]
		Pol κ	++++ [213] ++++ in cooperation with Pol ζ in vivo [222]	dAMP [213, 222]
		PrimPol	- [233]	
Alkylation	N3-me-A	Replicative Pols		
		Pol α	[82]	dAMP [82]
		Pol δ	[82]	
		Specialized Pols		
		Pol η	++++ [82]	dTMP ≈ dAMP [82]
		Pol ι	+ [82, 202]	dTMP ≈ dAMP [82]
		Pol κ	+++ [82, 202]	dTMP [82]
	O^6^-me-G	Replicative Pols		
		Pol α	+++ [90, 91]	
		Pol δ	+++ [17]; ++++ [87]	dCMP ≈ dTMP [87]
		Specialized Pols		
		Pol η	+ [90]; ++++ [87, 89]	dCMP ≈ dTMP [87, 89]
		Pol ι	++ [87, 203]	dTMP [87, 203]
		Pol κ	+++[87]	dCMP ≈ dTMP [87]
	εA	Replicative Pols		
		Pol δ	- [17, 100]	
		Specialized Pols		
		Pol η	+++ [100]	dTMP [100]
		Pol ι	++ [205, 206]; ++++ in cooperation with ScPol ζ [206]	dTMP with Mg^2+^ and dCMP with Mn^2+^ [205] dCMP ≈ dTMP [206]
		Pol κ	+ [100]	dTMP and deletions [100]

Replication efficiency: - – inhibition; + – low; ++ – incorporates nucleotides opposite the lesion but extension is inefficient; +++ – moderate; ++++ – high.

**Oxidized nucleobase derivatives**

**Fig. 1:**
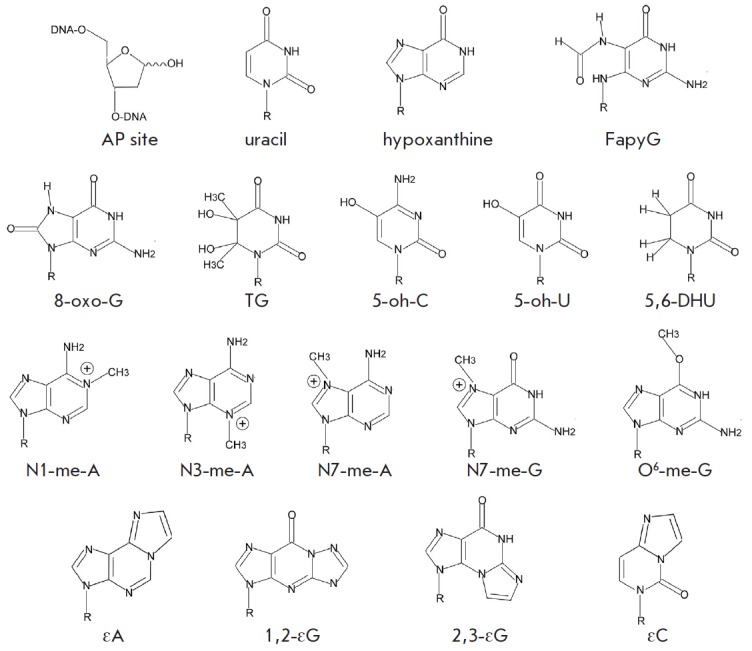
Common non-bulky DNA lesions

DNA bases are oxidized in cells when they interact with reactive oxygen species (ROS) formed by ionizing radiation or produced under physiological conditions. The frequency of ROS-induced damage in mitochondrial DNA is much higher than that of nuclear DNA [33]. Different ROS vary in their reactivity. The superoxide radical (O_2_•) and hydrogen peroxide (H_2_O_2_) are weakly reactive, while the hydroxyl radical (OH•) is extremely reactive and damages all four DNA bases; singlet oxygen (1O_2_) predominantly attacks guanine residues [34-36]. Oxidative stress is responsible for more than a hundred types of DNA lesions [34]. The most common and biologically relevant oxidized derivatives of nucleobases include 7,8-dihydro-8-oxoguanine (8-oxo-G), thymidine glycol (TG), 5-hydroxycytosine (5-oh-C), 2,6-diamino-4-hydroxy-5-formamidopyrimidine (FapyG), and 4,6-diamino-5-formamidopyrimidine (FapyA) (*Fig. 1*). Formamidopyrimidine lesions result from the opening of the imidazole ring caused by the attack of ROS [35, 37-39].

8-oxo-G and TG are among the most common DNA lesions induced by ROS. 1,000–2,000 8-oxo-G and up to 2,000 TG are formed daily in a single human cell [35]. 8-oxo-G is a highly mutagenic lesion. Most Pols incorporate opposite 8-oxo-G dAMP by forming Hoogsteen hydrogen bonds and giving rise to GC→TA transversions (*Table*) [40-42]. TG is a non-mutagenic but highly toxic lesion that suppresses the activity of replication enzymes (*Table*) [43, 44]. The mechanisms that reduce the mutagenic potential of oxidative nucleotide damage in cells are complex and include alternative variants of repair.

**DNA damage caused by deamination of DNA bases**

Loss of an amino group by DNA bases in the cell occurs either spontaneously [45, 46] or as a result of oxidative deamination induced by reactive nitrogen species (e.g., those formed by inflammation) [47-49] or is generated ezymatically. Deamination of cytosine residues by cytidine deaminases and the subsequent excision of uracil yielding AP sites play a crucial role in mutagenesis during the maturation of the variable regions of immunoglobulin genes in B lymphocytes in mammals [14, 15].

Single-stranded DNA (e.g., DNA being replicated or actively transcribed) undergoes deamination much more often (over 100 times) than the double-stranded DNA [50, 51]. Pyrimidine residues are more prone to spontaneous deamination than purines [51, 52]; however, purine bases undergo oxidative deamination more frequently [53]. Cytosine deamination that yields uracil is most common in the cell (*Fig. 1*). An average of 100– 500 cytosine residues are deaminated daily, generating uracil in a single mammalian cell [45, 51, 54]. Uracil derivatives, 5-hydroxyuracil (5-oh-U) and 5,6-dihydrouracil (5,6-DHU), can be formed by cytosine deamination and the simultaneous attack by free radicals or ionizing radiation (*Fig. 1*) [55, 56]. Genomic DNA also contains deamination products of adenine (hypoxanthine) and guanine (xanthine and oxanine) (*Fig. 1*). The frequency of hypoxanthine and xanthine formation is 1–7 per 106 nucleotides [57-59]. Deaminated DNA bases do not disrupt the functioning of eukaryotic Pols but have a high mutagenic potential, generating point mutations. Eukaryotic Pols incorporate dAMP opposite uracil, resulting in GC→AT transversions in the subsequent replication rounds (*Table*) [60]. Furthermore, dCMP is preferentially incorporated opposite hypoxanthine, causing the AT→GC transversion [61-63]. Xanthine and oxanine form hydrogen bonds with thymine, thereby causing GC→AT transversions during replication [64].

Deamination of 5-methylcytosine (5-me-C), which generates thymine and causes direct GC→AT transversions, also makes a significant contribution to DNA mutagenesis [45]. Although only 3% of cytosine residues in human genomic DNA is methylated, 70–80% of the GpG islands that suppress gene expression contain 5-me-C and, thus, act as mutagenesis hot spots in dividing mammalian cells [65, 66].

The lesions caused by deamination of DNA bases are predominantly repaired via the BER pathway.

**Alkylated nucleobase derivatives**

Exogenous alkylating agents are electrophilic compounds that exhibit an affinity for the nucleophilic centers of organic molecules and include a broad range of chemical agents which play a significant role in nucleobase alkylation. For example, exogenous alkylating agents are present in food as nitrosamines (N-nitrosodimethylamine and N-nitrosodiethylamine are formed by the interaction between amines and nitrites during smoking or intensive thermal treatment) [67, 68] and are found in the environment as haloalkanes (vinyl chloride used as a raw material in the plastics industry, agricultural fumigant bromomethane, and the coolant chloromethane) [69-71]. Some alkylating compounds, such as cyclophosphamide, melphalan, busulfan, and temozolomide, are widely used in chemotherapy [72, 73]. According to their nucleophilic substitution mechanism, alkylating agents can be subdivided into SN1-type (monomolecular substitution with an intermediate formation: nitrogen mustard, N-nitroso-N-methylurea) and SN2-type compounds (one-stage bimolecular substitution: methyl methanesulfonate, dimethyl sulfate, and busulfan) [74, 75].

All four DNA bases have been found to contain numerous potential sites of alkylation (N1, N3, N6, and N7 in adenine; N1, N2, N3, N7, and O6 in guanine; N3, N4, and O_2_ in cytosine; and N3, O_2_, and O4 in thymine); however, all these sites differ in reactivity. The common and biologically important alkylated nucleobase derivatives include N3-methyladenine (N3-me-A), O6-methylguanine (O^6^-me-G), 1-methyladenine (N1- me-A), N7-methylguanine (N7-me-G), and N2-ethylguanine (N2-et-G) (*Fig. 1*) [74-77]. The most common N-methylation products are N7-me-G and N3-me-A. N7-me-G may account for up to 70–80% of methylated DNA lesions.

Endogenous genotoxic agents also contribute to the alkylation of DNA bases. S-adenosylmethionine (SAM) is a weak alkylating agent that acts as a methyl group donor in cellular transmethylation reactions. Approximately 4,000, 600, and 10–30 residues of 7-me-G, 3-me-A and O^6^-me-G, correspondingly, [78, 79] are believed to be formed daily in mammalian cells in SAM-mediated reactions.

N3-me-A accumulation is cytotoxic, because it blocks replication due to the disruption of the contacts between the polymerase active site and the N3 atom of adenine in the minor groove of DNA (*Table*) [80-82]. Studies of the effect of N7-me-G on the functions of Pols are challenging because of the high instability of the damaged base. Methylated guanine residues do not inhibit Pol I function in *Escherichia coli* [83]. However, it has been recently demonstrated using a chemically stable N7-me-G analogue that human Pol β incorporates nucleotides opposite this lesion with low efficiency and fidelity (*Table*) [84]. N7-me-G can undergo spontaneous depurination to yield cytotoxic AP sites [75]. Furthermore, N7-me-G with the opened imidazole ring (me-Fapy-G) inhibits replication [85, 86]. O^6^-me-G is generated predominantly as a result of DNA exposure to SN1-type chemical agents [78]. This lesion exhibits mutagenic and carcinogenic properties, because it forms bonds with thymine and causes GC→AT transversions during replication [87-89]. O^6^-me-G can also suppress the function of certain Pols (*Table*) [90, 91]. Direct reversal repair by alkyltransferases and dioxygenases plays a crucial role in the repair of non-bulky alkylated nucelobases, along with BER [92].

Exocyclic nucleobase adducts with the etheno ring (1,N6-ethenoadenine (εA), 1,N2-ethenoguanine (1,2-εG), N2,3-ethenoguanine (2,3-εG), and 3,N4-ethenocytosine (εC)) can be classified as relatively non-bulky lesions and are also repaired by the enzymes involved in the repair of alkylated DNA bases (*Fig. 1*). The formation of these adducts is caused by aldehydes resulting from lipid peroxidation by oxygen and nitrogen free radicals [93, 94], as well as some genotoxic industrial chemicals (e.g., vinyl chloride and urethane) [95]. Exocyclic DNA lesions exhibit high mutagenic and genotoxic properties both *in vitro *and *in vivo* [96-99]. The 1,N6-etheno group disrupts the Watson-Crick interactions and suppresses the function of most Pols, including some specialized Pols (*Table*) [17, 100, 101].

## REPAIR OF NON-BULKY DNA LESIONS

**Base excision repair (BER)**

**Fig. 2:**
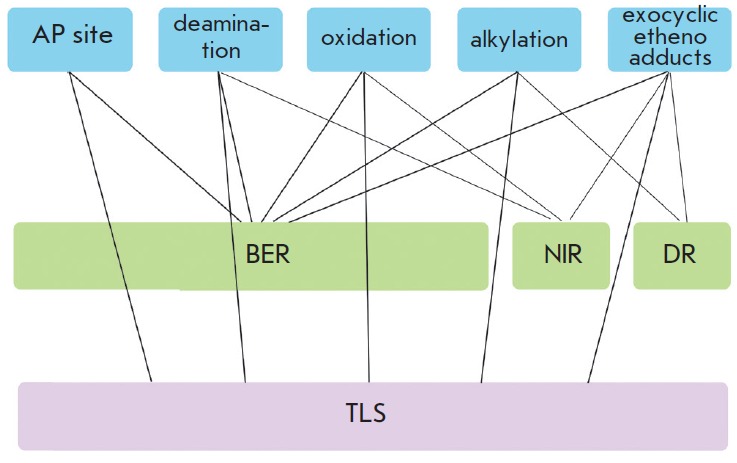
Basic pathways of non-bulky DNA lesions repair in humans

**Fig. 3:**
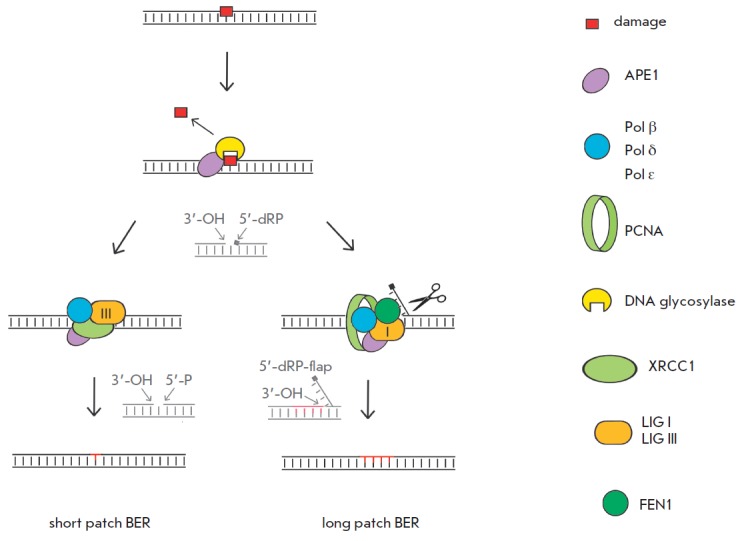
The short-patch and long-patch pathways of BER in humans

BER plays a key role in the elimination of non-bulky DNA lesions (*Fig. 2* and *Fig. 3*). BER includes two sub-pathways: the short-patch and long-patch BER. The short-patch BER replaces the lesion with a single nucleotide, while the long-patch BER excises 2–8 nucleotides [102].

The classic BER pathway consists of the following key steps: 1) elimination of a damaged base: damage recognition and cleavage of the N-glycosidic bond by a specific multifunctional DNA glycosylase, yielding an AP site; 2) hydrolysis of the phosphodiester bond at the 5’ end of the AP site by AP endonuclease, yielding 3’-OH and 5’-2-deoxyriboso-5-phosphate (5’-dRP); 3) excision of 5’-dRP and filling of the gap by a specialized Pol; and 4) ligation with DNA ligase (*Fig. 3*) [7-9, 102].

BER is initiated by DNA glycosylase. DNA glycosylases possessing only the glycosylase activity are known as monofunctional ones (e.g., uracil-DNA glycosylase UNG and N-methylpurine DNA glycosylase MPG, NEIL3) [103-105]. In this case, the AP site is cleaved by AP endonuclease APE1 [23, 106]. However, a number of DNA glycosylases simultaneously exhibit the DNA glycosylase and AP lyase activities: OGG1 (weak AP lyase activity), NEIL1, NEIL2, and NTH1. These DNA glycosylases are known as bifunctional: they excise a damaged base and hydrolyse DNA strands at the 3’ end of the AP site to form a 3’-α,β-unsaturated aldehyde group (3’-α,β-4-hydroxypentene- 2-al) (NTH1 and OGG1) or 3’-phosphate (NEIL1 and NEIL2) [103-105]. APE1 (3’-phosphodiesterase activity) and polynucleotide kinase/phosphatase (PNKP) (3’-phosphatase activity) are involved in the elimina tion of the 3’-aldehyde group and 3’-P, respectively [107-109].

In most cases, DNA lesions are repaired by the short-patch sub-pathway of BER. The XRCC1 protein (X-ray repair cross-complementing protein 1) plays a crucial role in the regulation of enzymatic activity during the short-patch BER and carries out structural and coordinating functions. [110, 111]. However, in some cases, BER occurs via the long-patch sub-pathway. In the latter case, the enzymatic functions are coordinated by the DNA clamp PCNA (proliferating cell nuclear antigen) and clamp loader RFC (replication factor C) [112]. The mechanisms that control the BER pathway selection have not been elucidated yet. The long-patch sub-pathway may be preferable in the S-phase in actively dividing cells [113] or when the excision of the 5’-dRP is impeded (e.g., for AP site analogues).

Pol β belongs to the X family of DNA polymerases and is a principal enzyme responsible for DNA synthesis during BER in mammals. Pol β simultaneously exhibits the 5’-dRP-lyase activity and can both efficiently fill the gap and excise the 5’-dRP formed after the AP site cleavage by APE1 [114]. Three other Pols, Pol λ, Pol ι, and Pol θ belonging to the X, Y, and A polymerase families, respectively, also retain the 5’-dRP-lyase activity and can hypothetically be involved in BER of some DNA lesions or play the role of Pol β-backup enzymes [115-118]. The high-fidelity replicative polymerases Pol δ and Pol ε can also be implicated in the long-patch BER [119]. Pol β, Pol δ, or Pol ε performs strand displacement synthesis for a DNA strand with a lesion. This produces the flap structure formed by three DNA strands; one of these strands has an overhanging single-stranded 5’-region, which is cleaved off by flap endonuclease FEN1 [120]. DNA ligase III (LIG3α) [121] and DNA ligase I (LIG1) [122] connect two DNA strands during the short- and long-patch BER sub-pathways, respectively.

There are more than 10 different human DNA glycosylases specialized in the recognition of various types of DNA lesions [103, 104]. A single lesion can often be recognized by several DNA glycosylases.

Uracil residues are predominantly eliminated by the monofunctional uracil-DNA glycosylases that yield AP sites during BER. Five uracil-DNA glycosylases have been identified in mammals, a fact that emphasizes the special role of cytosine deamination in mutagenesis and cytotoxicity. Two uracil-DNA glycosylase variants encoded by the *UNG* gene are known in humans: UNG1 and UNG2. The isoforms are generated by alternative splicing and reading transcripts from alternative promoters. UNG2 contributes to the repair of U:A lesions in nuclear DNA, while UNG1 is involved in the repair of uracil residues in mitochondrial DNA [123-125].

SMUG1 (single-stranded selective multifunctional uracil-DNA glycosylase 1) is likely a backup uracil-DNA glycosylase that also participates in the excision of 5-hydroxymethyluracil and oxidized pyrimidines [126, 127]. UNG1 and UNG2 excise uracil residues from single- and double-stranded DNA, while SMUG1 exhibits high activity on single-stranded DNA [128]. Mismatch-specific thymine-DNA glycosylase (TDG) and methyl-CpG-binding protein 4 (MBD4) participate in the repair of U and T mispaired with G, as well as in the repair of deaminated N5-me-C in CpG islands and, therefore, are involved in DNA demethylation and the epigenetic regulation of gene expression [129-132].

Oxidized nucleobase derivatives are predominantly repaired by bifunctional DNA glycosylases. OGG1 is the key DNA glycosylase that ensures the repair of 8-oxo-G during BER [133, 134]. Several OGG1 isoforms are generated by alternative splicing. Isoform 1a is mainly encountered in the nucleus, while isoform 2a – in mitochondria [135, 136]. Another DNA glycosylase, NEIL1, is also involved in the repair of 8-oxo-G, although its activity is much less pronounced [137, 138].

The DNA glycosylases NTH1 and NEIL1 participate in the repair of various oxidized derivatives of pyrimidine nucleotides [138-143]. NEIL2 is involved in the repair of oxidized cytosine lesions (5,6-DHU, 5-oh-U, and 5-oh-C) [144]. NEIL3 can recognize and excise various oxidized base derivatives, including TG and Fapy-purine lesions; however, its functions remain insufficiently understood [145, 146]. OGG1 and NTH1 excise lesions from double-stranded DNA, while NEIL1, NEIL2, and NEIL3 function efficiently on single-stranded DNA templates and are possibly involved in the repair of oxidized nucleobases during replication and transcription [147-150].

N-methylpurine DNA glycosylase (MPG), also known as N-alkyladenine DNA glycosylase (AAG) and 3-methyladenine DNA glycosylase (MAG), is involved in the repair of alkylated purine bases during BER. MPG is characterized by a broad substrate specificity as it recognizes and excises N3-me-A, N7-me-G, N1- me-T, εA, and 1,2-εG [151-155]. In addition to repairing alkylated bases, MPG is also involved in the repair of DNA damage caused by deamination of purine bases (hypoxanthine, xanthine, and oxanine) [152, 153, 156, 157]. The structural features of the active sites of DNA glycosylases that guide the recognition of various lesions were discussed in a previous review [104].

**BER of nucleotides paired with damaged DNA bases**

Interesting mechanisms of DNA damage repair preventing mutagenesis involve mismatch-specific DNA glycosylases that excise undamaged bases paired with damaged nucleotides. For example, MUTYH, an adenine DNA glycosylase, recognizes adenine paired with 8-oxo-G [158, 159]. The excision and substitution of dAMP with the complementary dCMP prevents transversions in the subsequent replication rounds. The repair of the A:8-oxo-G base pair was reconstituted *in vitro *with MUTYH, APE1, Pol λ, and DNA ligase I in the presence of PCNA, RPA, and FEN1 [159]. DNA glycosylase TDG is involved in a similar mechanism of excision of thymine paired with noncomplementary cytosine and guanine bases containing the exocyclic etheno ring [160].

**Nucleotide incision repair (NIR)**

Nucleotide incision repair (NIR) is an alternative pathway for lesion excision that requires AP endonucleases (*Fig. 2*) [161-162]. In this process, the excision of a damaged nucleotide does not involve DNA glycosylases and does not require the formation of a potentially mutagenic intermediate product – AP site. APE1 cleaves DNA at the 5’ end of the damaged nucleotide and leaves the undamaged 3’ end free for Pol. The remaining damaged nucleotide can be subsequently excised by flap endonuclease FEN1. Pol β and LIG1 fill the gap and join segments of DNA *in vitro *[163]. The role of NIR was initially demonstrated for oxidized DNA nucleotides. APE1 can recognize and be involved in the excision of TG, 5,6-dihydropyrimidines and 5-hydroxypyrimidines [161, 164]. It has been recently demonstrated that NIR could also be an alternative pathway for the repair of other non-bulky lesions, such as uracil [165], εA, and εC [164].

**Direct reversal repair (DRR or DR)**

Several types of non-bulky lesions can be also repaired by enzymes through direct reversal (*Fig. 2*). These enzymes include the AlkB family of dioxygenases (oxidative demethylases) and alkyl transferases, which participate in the repair of alkylated DNA bases [166]. Oxidative demethylation of damaged bases catalyzed by dioxygenases proceeds through the Fe(II)-dependent mechanism of alkyl groups oxidation with molecular oxygen [167]. Eight homologues of *E. coli *AlkB (ALKBH1-8) were identified in humans. The dioxygenases ALKBH2 and ALKBH3 play a key role in the demethylation of N1-me-A, N3-me-C, εA, and alkylated thymine bases [168-170].

Human O6-alkylguanine-DNA alkyltransferase (AGT or MGMT) is involved in the repair of O^6^-me-G and O4-me-T; it also recognizes and excises a number of relatively bulky alkyl groups in O6-modified bases [171-173]. AGT irreversibly binds and transfers the methyl group using the thiol group of cysteine as an acceptor (the SN2 mechanism) [174, 175].

## DNA TRANSLESION SYNTHESIS

**Fig. 4:**
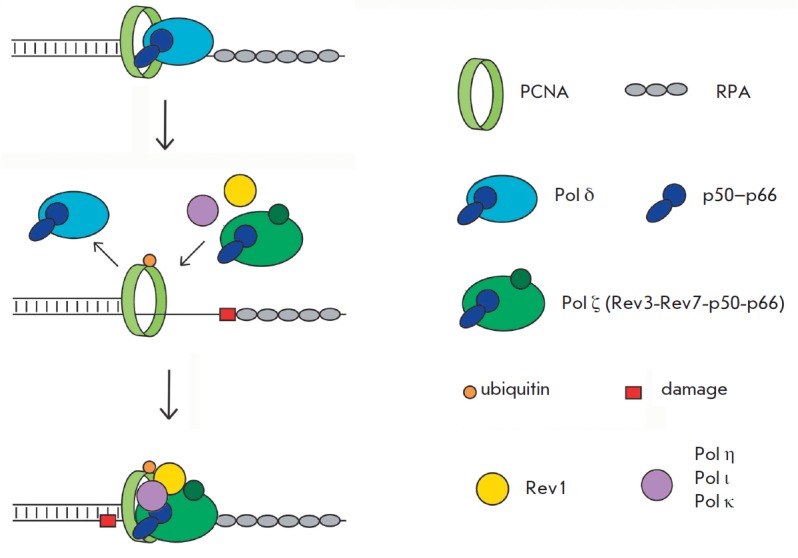
DNA translesion synthesis by specialized DNA polymerases in humans

Some DNA lesions cannot be rapidly repaired. Persistent DNA damage disrupts the functions of the high-fidelity replicative Pol α, Pol δ, and Pol ε (*Table*) and interrupts replication, resulting in cell-cycle termination, chromosomal instability, or cell death. Recruitment of specialized Pols, belonging to various families and specializing in the replication of various lesions, to the stalled replication fork is the key mechanism of progression through the replication blocks (*Fig. 2* and *Fig. 4* and *Table*). Y-family Pol ι, Pol η, Pol κ, and Rev1 and B-family Pol ζ play a crucial role in replication through non-bulky DNA lesions in human cells [176-179]. To function efficiently, specialized Pols form multisubunit complexes (mutasomes or translesomes) consisting of Pols and proteins that possess structural and regulatory functions and are involved in coordinating the activity of the complex (*Fig. 4*). Specialized Pols possess active sites lacking strict structural requirements to the DNA template and efficiently incorporate nucleotides opposite lesions. Due to the tolerance of the active site, non-canonical hydrogen bonds utilization during base pairing, and the absence of the 3’→5’ proofreading ac tivity, these enzymes are characterized by low fidelity of DNA synthesis, leading to mutagenesis in the organism [180].

**Role of Pol ι, Pol η, and Pol κ in DNA translesion synthesis**

Y-family Pol ι, Pol η, and Pol **κ **are single-subunit enzymes characterized by very low-fidelity of synthesis (10^-1^–10^-4^) and low processivity [181-186]. Pol η and Pol ι incorporate only one or several nucleotides opposite a damaged site and function as inserters. The primary role of Pol η in the cell is to accurately and efficiently replicate through photoproducts (thymine–thymine cyclobutane dimers) and to protect cells against UV radiation [187, 188]. Nevertheless, Pol η efficiently incorporates nucleotides opposite some other non-bulky lesions (*Table*) [82, 89, 187, 189-196]. For example, Pol η shows high efficiency of synthesis opposite AP sites, incorporating dAMP and dTMP [189, 196], as well as opposite oxidized nucleobases preferentially incorporating correct nucleotides opposite 8-oxo-G and TG [192, 193, 195], and thereby playing a key role in the protection of cells against the most common cytotoxic and mutagenic lesions.

Pol ι efficiently incorporates nucleotides with different accuracies opposite a number of non-bulky DNA lesions, such as AP sites [189, 196-199], uracil and its derivatives [200], 8-oxo-G [201], N3-me-A [190, 202], O^6^-me-G [203], 1,2-εG [204], and εA [205, 206] (*Table*). The ability of Pol ι to form non-canonical hydrogen bonds during nucleotide base pairing plays an important role in efficient translesion DNA synthesis. For example, Pol ι utilizes Hoogsteen interactions when incorporating nucleotides opposite εA with the etheno ring that blocks the formation of Watson-Crick hydrogen bonds [206]. Pol ι was also shown to use Hoogsteen interactions to incorporate dNMP opposite O^6^-me-G, whose methyl group is exposed in the major groove [203].

An unusual feature of Pol ι is the preferential incorporation of dGMP opposite thymine, uracil, and uracil derivatives [184, 185, 200, 207]. This property possibly plays an important role in reducing the mutagenic potential of deaminated cytosines and 5-me-C [200]. The incorporation of dGMP opposite T in templating DNA is stabilized by the unique hydrogen bond that is formed directly between the N2 atom of dGTP and the Gln59 residue in the active site of Pol ι [208]. Pol ι efficiently incorporates nucleotides opposite AP sites, and it is one of the few Pols that preferentially incorporate either dGMP or dTMP, rather than dAMP opposite this lesion [189, 196, 198, 199].

The main cellular function of Pol κ is DNA synthesis past guanine deoxyribonucleotide adducts with chemical groups at the N2 position, which are formed under exposure to some carcinogens. These adducts include both bulky [209-211] and relatively non-bulky lesions (1,2-εG and 2,3-εG) (*Table*) [96, 212]. Pol κ is also involved in efficient and accurate replication past TG [213].

Pol η and Pol ι are not efficient in primer elongation after nucleotide incorporation opposite a DNA lesion. Therefore, further DNA synthesis including elongation from mispaired primer termini is carried out by the Pol-extender. Unlike Pol η and Pol ι, Pol κ efficiently extends mispaired primer termini [214, 215]. Possibly, it can act as an extender in some cases and contribute to the fixation of mutations. However, the key role in the extension step during DNA translesion synthesis is played by the B-family DNA polymerase Pol ζ [197, 216].

**Role of Pol ζ and Rev1 in DNA translesion synthesis**

Pol ζ consists of four subunits: the catalytic Rev3 and regulatory Rev7, p50, and p66 subunits [217-219]. The four-subunit human DNA polymerase ζ complex was isolated in 2014 [218]; however, DNA translesion synthesis of non-bulky DNA lesions involving human Pol ζ is yet to be studied. *S. cerevisiae *Pol ζ carries out efficient extension of mispaired primer termini and primers paired with lesions [215, 220].

It has also been demonstrated that yeast Pol ζ cooperates with human Pol ι or yeast Pol η for efficient replication through AP sites [215, 221], with Pol κ for accurate replication past TG [222] and Pol ι for efficient replication opposite εA [206]. Unlike Y-family Pols whose functions are interchangeable, the loss of Pol ζ catalytic activity in mammalian cells is lethal, which is indicative of its role in the replication of a large number of endogenous DNA lesions [223, 224].

Another protein belonging to the Y family, Rev1, exhibits weak DNA polymerase activity, as it preferentially incorporates dCMP opposite template G but plays key structural and regulatory roles in mutasome assembly [177]. Rev1 contains binding sites for both the Y-family Pol ι, Pol η, and Pol κ (via the RIR motif in Pol ι, Pol η, and Pol κ) [225-227] and several Pol ζ subunits [228-230]. Rev1 interacts with the nonubiquitinylated and mono-ubiquitinylated PCNA processivity factor [231, 232]. The presence of multiple binding sites for Pols and replication factors allows to coordinate the activity of replication enzymes and timely ensures DNA synthesis by switching from the high-fidelity Pols to specialized Pols, and from the Y-family Pol-inserter to the processive Pol ζ (*Fig. 4*). However, the detailed mechanism of mutasome operation within the framework of the two-polymerase replication model has not been completely elucidated.

**The role of PrimPol in DNA translesion synthesis**

In 2013, a new type of specialized human Pol was described: primase-polymerase PrimPol. It simultaneously exhibits DNA polymerase and primase activities but differs from the Pol α-primase complex in its ability to initiate DNA synthesis using dNMP [233-235]. PrimPol does not belong to any of the families of the known eukaryotic Pols but belongs to the AEP family of primases [236]. *PRIMPOL *knockout sensitizes cells to DNA lesions [233] and slows down the replication fork progression in the absence of exogenous damaging factors [237]. PrimPol efficiently incorporates nucleotides opposite several non-bulky DNA lesions (e.g., accurate synthesis opposite 8-oxo-G) (*Table*) [234, 238]. However, re-initiation of replication downstream of DNA damage is believed to be the key function of PrimPol [239]. Unlike other human Pols, the activity of PrimPol is not stimulated by PCNA [240] but is activated by the PolDIP2 protein [241]. PrimPol has been detected in both the nucleus and mitochondria [234] and is activated by the mitochondrial helicase Twinkle [242].

## CONCLUSIONS

In the course of evolution, living organisms have developed a machinery that efficiently protects them against the genotoxicity of DNA damage. It includes mechanisms for removing DNA damage and restoring the original DNA structure (repair), as well as mechanisms that ensure cell tolerance to DNA damage without removing them (DNA translesion synthesis). BER and DNA glycosylases with a different substrate specificity to DNA lesions play a crucial role in the repair of non-bulky DNA lesions. In recent years, alternative mechanisms for the repair of non-bulky DNA lesions have been discovered (such as NIR, direct reversal repair with dioxygenases, and APE1-independent repair of AP-sites). The cellular repair pathways have been shown to overlap and duplicate in their functions. The small number of lesions that remain in genomic DNA often block high-fidelity replicative Pols and lead to a switch to replication with specialized Pols. Recently, a new mechanism for overcoming blockages in replication caused by DNA damage has been discovered. It relies on DNA polymerase and primase PrimPol, which re-initiates replication downstream of DNA damage. The diversity of the mechanisms of DNA repair and DNA translesion synthesis provides high protection against the cytotoxic and mutagenic effects of DNA damage in cells.

Accumulation of non-bulky lesions as a result of disrupted functions of reparative/replicative enzymes leads to the development of human diseases, such as cancer. The link between the functions of reparative/ replicative enzymes and human diseases has been dis cussed in reviews [243-246]. The search for efficient methods to regulate the activity of the enzymes involved in repair and replication is a promising strategy that could give rise to novel therapeutic approaches.

## Abbreviations

AP sites: apurinic/apyrimidinic sites
BER: base excision repair
ROS: reactive oxygen species;
5’-dRP: 5’-2-deoxyribose-5-phosphate
DRR: direct reversal repair
NER: nucleotide excision repair
NIR: nucleotide incision repair
FapyA: 4,6-diamino-5-formamidopyrimidine
FapyG: 2,6-diamino-4-hydroxy-5-formamidopyrimidine
5,6-DHU: 5,6-dihydrouracil
5-oh-U: 5-hydroxyuracil
8-oxo-G: 7,8-dihydro-8-oxoguanine
5-oh-C: 5-hydroxycytosine
N1-me-A: N1-methyladenine
N3-me-A: N3-methyladenine
N5-me-C: 5-methylcytosine
N7-me-G: N7-methylguanine
O^6^-me-G: O6-methylguanine
SAM: S-adenosylmethionine
N2-et-G: N2-ethylguanine
TG: thymidine glycol
εA: 1,N6-ethenoadenine
1,2-εG: 1,N2-ethenoguanine
2,3-εG: N2,3-ethenoguanine
εC: 3,N4-ethenocytosine
